# Periodic TiO_2_ Nanostructures with Improved Aspect and Line/Space Ratio Realized by Colloidal Photolithography Technique

**DOI:** 10.3390/nano7100316

**Published:** 2017-10-12

**Authors:** Loïc Berthod, Olga Shavdina, Isabelle Verrier, Thomas Kämpfe, Olivier Dellea, Francis Vocanson, Maxime Bichotte, Damien Jamon, Yves Jourlin

**Affiliations:** 1 Lyon, UJM-Saint-Etienne, Laboratoire Hubert Curien UMR 5516, CNRS, Institut d’Optique Graduate School, F-42023 Saint-Etienne, France; loic.berthod@univ-st-etienne.fr (L.B.); olga.shavdina@univ-st-etienne.fr (O.S.); isabelle.verrier@univ-st-etienne.fr (I.V.); francis.vocanson@univ-st-etienne.fr (F.V.); maxime.bichotte@univ-st-etienne.fr (M.B.); damien.jamon@univ-st-etienne.fr (D.J.); Yves.Jourlin@univ-st-etienne.fr (Y.J.); 2 Laboratoire des Composants pour le Conversion de l’Energie (L2CE), Laboratoire d’Innovation pour les Technologies des Energies Nouvelles et des nanomatériaux (CEA/LITEN), F-38054 Grenoble, France; olivier.dellea@cea.fr

**Keywords:** sol-gel, TiO_2_, sub-wavelength structures, colloidal photolithography

## Abstract

This paper presents substantial improvements of the colloidal photolithography technique (also called microsphere lithography) with the goal of better controlling the geometry of the fabricated nano-scale structures—in this case, hexagonally arranged nanopillars—printed in a layer of directly photopatternable sol-gel TiO_2_. Firstly, to increase the achievable structure height the photosensitive layer underneath the microspheres is deposited on a reflective layer instead of the usual transparent substrate. Secondly, an increased width of the pillars is achieved by tilting the incident wave and using multiple exposures or substrate rotation, additionally allowing to better control the shape of the pillar’s cross section. The theoretical analysis is carried out by rigorous modelling of the photonics nanojet underneath the microspheres and by optimizing the experimental conditions. Aspect ratios (structure height/lateral structure size) greater than 2 are predicted and demonstrated experimentally for structure dimensions in the sub micrometer range, as well as line/space ratios (lateral pillar size/distance between pillars) greater than 1. These nanostructures could lead for example to materials exhibiting efficient light trapping in the visible and near-infrared range, as well as improved hydrophobic or photocatalytic properties for numerous applications in environmental and photovoltaic systems.

## 1. Introduction

Colloidal photolithography [1,2] has several advantages, the most important one being its ability to periodically nano-structure large surfaces which can be planar or non-planar (curved of cylinder based shape). The method uses microspheres arranged in a regular grid to focus light into a photosensitive material. It is based on a 2D hexagonal self-arrangement of the microspheres in a monolayer. The concentration of the optical field underneath the microspheres called ‘photonic nanojet’ can illuminate the photosensitive layer locally, leading to a latent image according to the arrangement of the microspheres, which is then chemically developed.

Among the photosensitive materials, TiO_2_ sol-gel material is attractive because of its optical and chemical properties [3], especially when it is nanostructured [4]. TiO_2_ is well known for its high refractive index (up to 2.2 in its anatase phase), for its high mechanical and chemical stability, as well as for its photocatalytic properties. Association of the colloidal photolithography with TiO_2_ material leads to innovative components that could be used for example in attractive environmental applications [5,6] as well as in the domain of solar and photovoltaic energy [7,8,9]. Combining colloidal lithography and direct photopatternable sol-gel TiO_2_ material leads to a unique and powerful technology allowing to perform microstructuring in only one technological step, without etching process, while being compatible with standard and non-standard large substrates.

When TiO_2_ sol-gel material, being a negative photoresist, is periodically structured by colloidal lithography, the nanojets issued from each microsphere will lead to the origination of periodic nanopillars [10]. One limit of this process according to the state of the art is a low aspect ratio (height/lateral size) of the achieved nanopillars (or rods) due to the shape of the nanojet inside the TiO_2_. This is unfortunate since very high nanopillars have interesting properties, for instance, with regards to hydrophobicity. In the present study this limit was exceeded, with nanopillars of up to several hundreds of nanometers in height compared to the commonly achieved several tens of nanometers. This was achieved by depositing the sol-gel material on a reflecting substrate, like aluminum, and exploiting standing wave effects between the different materials. This approach of using metal layers to confine and form the electric field has been successfully applied for other lithographic techniques (e.g., two-beam lithography [11]) but, to the best of the author’s knowledge, this approach is new to colloidal lithography.

A second optimization of the form of the nanojets promises to result in wider than usual nanopillars, increasing the line/space ratio (size of the pillar/period of the grating). Wide nanopillars can, for example, increase the absorption of light in the UV region and can lead to useful photo-catalysis phenomena as well as higher efficiency in solar cells. The idea is to apply a tilt to the incident wave focused into the TiO_2_ material. Some authors have already considered microsphere photolithography under oblique incidence to produce arbitrary nano-patterns by projection of a pixelated optical mask into the photoresist [12]. In comparison, our approach is simpler because it is based on exposure of the TiO_2_ sol-gel directly through a mask of a SiO_2_ microspheres monolayer without any other intermediary optical system.

In the following, the results of rigorous simulations of the optical field behind the microspheres are presented, followed by experimental demonstrations of the two mentioned approaches for achieving wider nanopillars with complex shapes, as well as very high aspect ratio columns.

## 2. Materials and Methods

### 2.1. Rigorous Optical Simulation of Nanojets Created by Microsphere Arrays

In order to predict the distribution of the electric field from the array of microspheres, the geometry of the elementary cell of a hexagonal grating of silica microspheres was defined using a MATLAB routine for later use in an rigorous coupled-wave analysis (RCWA) [13] based optical propagation code (“MCGratings” [14]), which allows the calculation of the electromagnetic field distribution behind the microspheres. Each microsphere of 1 µm diameter was longitudinally (i.e., in the direction of light propagation) discretized in 35 layers, which is a good compromise for determining a sufficiently exact representation of the electromagnetic field in an acceptable calculation time. The number of considered Fourier-orders was determined by repeatedly calculating several representative structures with an increasing number of Fourier-orders and verifying the convergence of the results towards a solution that is sufficiently stable. 15 × 25 Fourier-orders were used for the calculation in this paper, as they proved to be a good compromise between calculation time and the required precision of the result.

### 2.2. Direct Photolithography of the Sol-Gel TiO_2_

In order to prepare TiO_2_-based photoresist, specific sol-gel formulations were used. The final sol is prepared from titanium isopropoxide orthotitanate (TIPT) complexed by benzoyl acetone (BzAc), using a mixture of two different primary sols as detailed in [4]. The so-obtained final sol can be coated on glass substrates by spin-coating and is thus compatible with large sized substrates. The deposited sol-gel TiO_2_ film is coated by a microspheres monolayer using the Langmuir Blodgett method that leads to hexagonal self-organization of the particles, as detailed in [10]. Further chemical and optical properties of the sol-gel and details about its preparation, as well as required parameters and details about the photolithographic patterning process can be found in previous works by the authors [4,10].

## 3. Results and Discussion

### 3.1. Simulation and Optimization of the Microsphere-Created Nanojets

**Figure 1:**
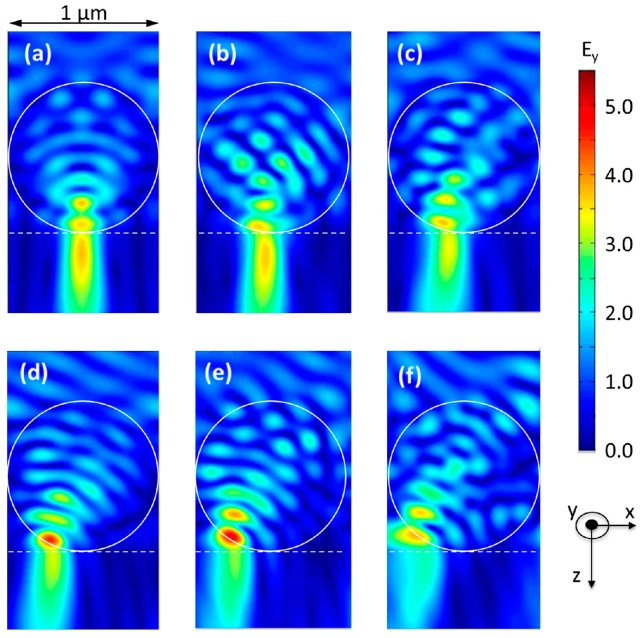
Mapping of the *E*_y_ component of the electric field for *λ* = 365 nm at different incidence angles: (**a**) 0°; (**b**) 5°; (**c**) 12°; (**d**) 20°; (**e**) 25° and (**f**) 30°.

The result of the optical simulations are presented in Figure 1, showing the obtained mapping of the component *E*_y_ of the electric field for a linearly polarized incident wave at the wavelength *λ* = 365 nm (corresponding to the i-line of the used narrow-band gas discharge mercury vapor lamp) propagating along the *z*-axis from air (top) to the sol-gel layer (down) with varying tilting angle, using optical borosilicate-crown glass (BK7) as substrate. The influence of the polarization direction of the incident light was found to be negligible for the overall structure of the field distribution, it was therefore fixed to having the E-Field along the *y* direction. In the simulation, the sol-gel layer thickness is supposed to be infinite underneath the microspheres that are arranged hexagonally with a period of 1 µm in *x*-direction, corresponding to their diameter. The incidence angle in *x*-direction of the incident wave varies from 0° (Figure 1a) to 30° (Figure 1f). The refractive index and the absorption of the sol-gel depend weakly on the illumination parameters and are thus difficult to fix in the modeling. As those changes are minor [10], we restricted for the scope of this paper the simulation to a non-absorbing sol-gel with a fixed refractive index of *n* = 1.63, measured by ellipsometry.

**Figure 2:**
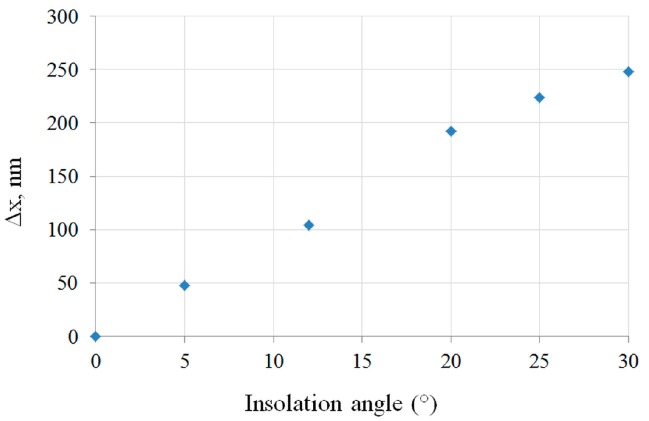
Lateral shift of the maximum intensity of the nanojet inside the TiO_2_ film versus exposure angle.

Having calculated the electric field, several parameters were studied to fully analyze and exploit the properties of the resulting nanojet. These parameters include the distance from the microsphere’s output face to the maximum intensity inside the nanojet, the nanojet’s length and diameter as well as the ratio between them, and the energy of the nanojet outside of the microsphere. The analysis of these parameters permits to choose the best exposure angle in order to obtain a required geometry (the length/width ratio and the shift of the nanojet along *x*-axis) with sufficient field concentration to expose the TiO_2_ in a reasonable time. It turns out that the overall shape and the length/width ratio of the nanojet remains stable up to tilting angles of about 25°, allowing for a flexible use of tilted incidence waves to change the shape of the developed structures. As expected, increasing the exposure angle shifts the lateral position of the maximal field amplitude laterally (Figure 2).

### 3.2. First Optimization: Increasing the Nanopillars Width and Creating Variable Shapes

**Figure 3:**
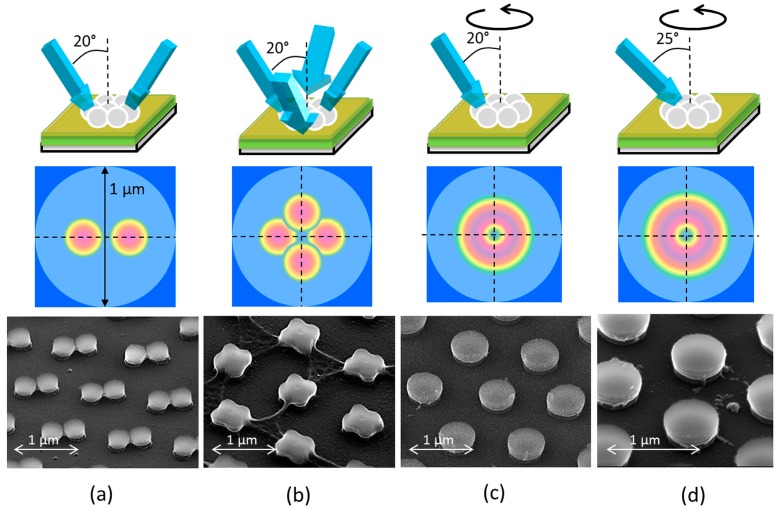
Illustration of the illumination conditions (top row), top view the expected nanojet arrangement in the TiO_2_ layer (middle row, red indicates increased intensity) and SEM photographs of the resulting TiO_2_ pattern (bottom row) of hexagonally periodic structures on BK7. (**a**) two opposite exposures of angle 20° leading to a bow-tie structure, (**b**) four exposures of angle 20° leading to clover leaf structure, (**c**) one exposure under an angle of 20° with substrate rotation leading to nanopillars of 500 nm diameter and (**d**) one exposure under an angle of 25° with substrate rotation leading to nanopillars of 600 nm diameter.

Figure 3 shows the different investigated optical setups and the resulting structures, comprising two exposures (Figure 3a) and four exposures (Figure 3b) under 20° exposure angle, as well as a continuous exposure at 20° (Figure 3c) and 25° (Figure 3d) inclination coupled to a continuous rotation of the substrate. According to Figure 2b for an angle of illumination of 20°, the maximum intensity shifts about 200 nm in the lateral direction *x* leading to different shapes on the illuminated area according to the three configurations shown in the middle row of Figure 3. After development in ethanol, each condition of illumination leads to different shapes of the resulting hexagonally periodic structure illustrated by the SEM photographs at the bottom row of Figure 3. For the two-beam case of Figure 3a a bow-tie pattern appears, the four exposures case of Figure 3b creates a clover-shaped pattern, whereas the rotation under an angle of 20° leads to large nanopillars with approximately 500 nm diameter (Figure 3c), and close to 600 nm diameter for 25° exposure angle, corresponding to a line/space ratio greater than 1 (Figure 3d).

### 3.3. Second Optimization: Increasing the Nanopillar’s Height

**Figure 4:**
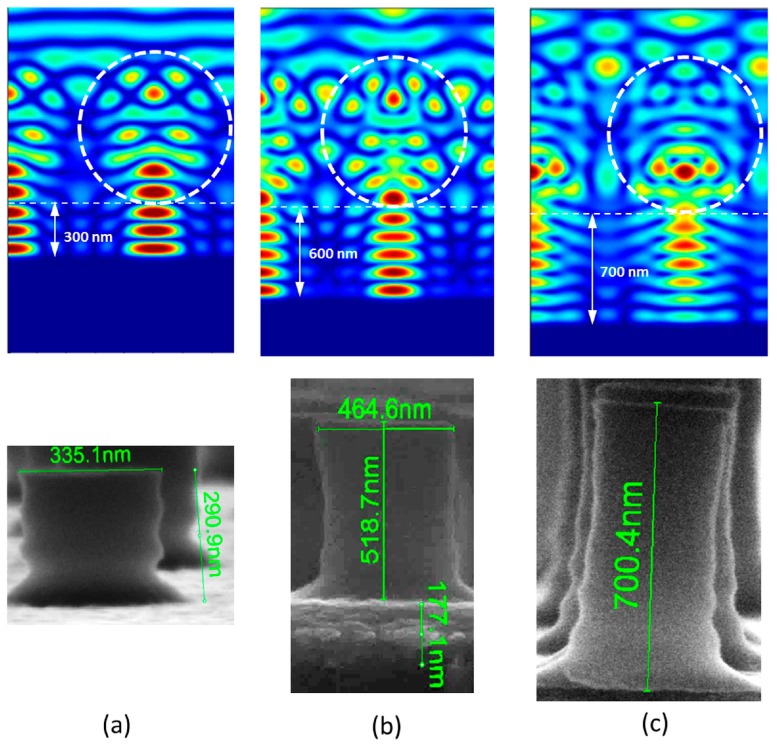
Simulation mapping of the electric field and SEM of the corresponding nanopillar with TiO_2_ initial film thickness of: (**a**) 300 nm; (**b**) 600 nm; and (**c**) 700 nm.

The second optimization of the geometry of the nanopillars, where the goal was to increase the pillar’s height, consists in using a reflecting substrate like, in the present case, a BK7 glass coated with a thin film (170 nm) of aluminum. As previously mentioned, the microspheres are deposited on the sol-gel TiO_2_ film. At normal incidence, the nanojet coming from the microspheres is reflected back, leading to a standing wave pattern which is notably longer than the nanojet without reflection, thus allowing higher nanopillars to be created. However, the standing wave creates intensity minima and maxima along the *z*-axis, which can be detrimental to the formation of smooth nanopillars if the sidewalls follow this modulation. Furthermore, at the interface TiO_2_-Al, the electric field value must be 0 in order to satisfy the continuity condition of its transverse component. This condition is not favorable for the stability of the structure because exposure of the TiO_2_ at the interface will be particularly ineffective. The distance *L* between two successive minima is related to the real part of the refractive index *n* of the film and to the insolating wavelength λ by *L* = *λ*/2*n*. For TiO_2_ of refractive index *n* = 1.84 at the insolating wavelength *λ* = 365 nm, *L* takes the value of 100 nm. The number of nodes is given by the film thickness divided by the distance *L*. For example, for a TiO_2_ film of thickness 600 nm, the nodes number is 6, as confirmed in the simulation shown in Figure 4b (top row).

Using the same experimental process as in the first part of this section but without any tilt, the nanopillar created by the nanojet reveals a shape in Figure 4b (bottom row) that is not so far from that predicted by the simulation. However, a smoothing effect of the exposed image is present, consolidating the pillars at their basis and also avoiding a too important modulation of the sidewalls. The smoothing can be attributed to the spectral linewidth of the illumination (which is neglected in the simulation), leading to a minor position change of the maxima and minima of the intensity and thus slightly blurring the interference pattern. Furthermore, a change of the refractive index and the absorption of the sol-gel can occur during exposure (bleaching effect, densification [10]) which will dynamically alter the field distribution and thus also create blurring. A detailed analysis of the importance of those effects is beyond the scope of this paper, the good agreement between simulation and results however confirm the eligibility of the modelling. The obtained pattern is 465 nm wide and 520 nm high with an under layer of thickness 177 nm. The addition of the height of the nanopillar and of the under-layer’s thickness corresponds to the initial TiO_2_ thickness deposited onto the substrate. The aspect ratio (height/width) is in this case equal to 1.2, but it has been improved with other samples when using a thicker film of TiO_2_ as shown below. Figure 4 shows the simulation results and the SEM images for different thicknesses of the TiO_2_ layer. For a thickness of 300 nm (Figure 4a), we can notice undulations on the nanopillars’ edge that follows the three nodes of the standing wave. For the higher nanopillars, the effect is less visible because of a reduced contrast between minima and maxima of the standing wave. In the case of a 300 nm thickness TiO_2_ film, the aspect ratio is 0.87 and is, as expected, smaller than the one obtained previously for a 600 nm thick film.

**Figure 5:**
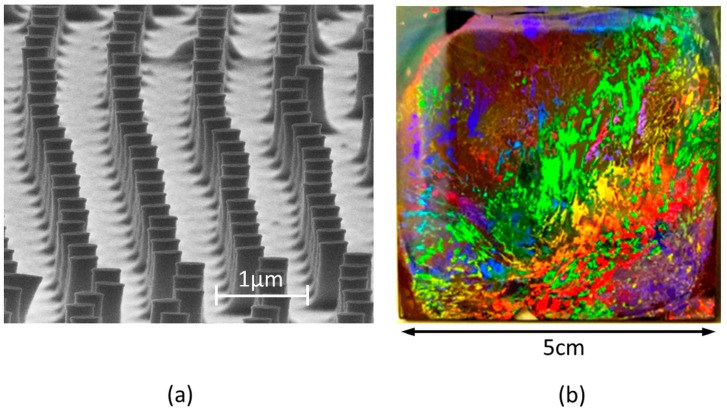
Global view of the hexagonally arranged nanostructures with high aspect ratio: (**a**) SEM of the array of nanopillars; (**b**) macroscopic view of the structure under white light illumination, showing the typical rainbow effect of periodic surface structures.

However, using even thicker TiO_2_ film, the height of nanopillars can reach 700 nm (Figure 4c) and the aspect ratio is 2.18. The SEM photographs of the hexagonal arrangement of the nanopillar are presented in Figure 5a. The diffraction effect shown in Figure 5b confirms the presence of the structuration on the whole surface. It also shows that the size of the perfectly crystalline regions of the surface is limited, in our case from several hundreds of µm to some mm, which is a known effect for Langmuir–Blodgett type monolayers [15].

## 4. Conclusions

In conclusion, the colloidal lithography technique is used to create original TiO_2_ nano-structures with high aspect ratio or with large line/space ratio, opening up its use for new applications. This study has demonstrated both theoretically and experimentally that the shape of the photonic nanojet limiting the nano-plots height can be modified using field enhancement by wave reflection and tilted illumination. The aspect ratio of the nanopillars was improved by 147% when using reflecting aluminum substrates compared to the case of transparent substrates. In this case, the shape of the pillars is additionally no longer trapezoidal, which is characteristic for transparent substrates, but approximates a square shape. Furthermore, it has been shown that the nanopillar’s width can be increased by employing multiple illumination technique and substrate rotation under oblique incidence, resulting in line/width ratios of the nanostructure grating larger than 1, strongly increased in comparison to the case of normal incidence. These improvements provide much flexibility to the colloidal lithography technique regarding the geometry of the microscopic structures, paving the way for a more widespread application to large scale planar and non-planar substrates.
